# Cell death mechanisms induced by synergistic effects of halofuginone and artemisinin in colorectal cancer cells

**DOI:** 10.7150/ijms.66737

**Published:** 2022-01-01

**Authors:** Rui-Hong Gong, Da-Jian Yang, Hiu-Yee Kwan, Ai-Ping Lyu, Guo-Qing Chen, Zhao-Xiang Bian

**Affiliations:** 1 Centre for Cancer and Inflammation Research (CCIR), School of Chinese Medicine, Hong Kong Baptist University, Kowloon Tong, Hong Kong S.A.R., China; 2 Chongqing Academy of Chinese Materia Medica, Chongqing 400065, China; 3 State Key Laboratory of Chinese Medicine and Molecular Pharmacology (Incubation), Shenzhen Research Institute, The Hong Kong Polytechnic University, Shenzhen 518057, China; 4 Research Centre for Chinese Medicine Innovation, The Hong Kong Polytechnic University, Hung Hom, Hong Kong S.A.R., China; 5 Department of Applied Biology & Chemical Technology, The Hong Kong Polytechnic University, Hung Hom, Hong Kong S.A.R., China

**Keywords:** halofuginone, artemisinin, synergy, colorectal cancer, apoptosis, autophagy

## Abstract

Our previous study found that the combination of halofuginone (HF) and artemisinin (ATS) synergistically arrest colorectal cancer (CRC) cells at the G1/G0 phase of the cell cycle; however, it remains unclear whether HF-ATS induces cell death. Here we report that HF-ATS synergistically induced caspase-dependent apoptosis in CRC cells. Specifically, both *in vitro* and *in vivo* experiments showed that HF or HF-ATS induces apoptosis via activation of caspase-9 and caspase-8 while only caspase-9 is involved in ATS-induced apoptosis. Furthermore, we found HF or HF-ATS induces autophagy; ATS can't induce autophagy until caspase-9 is blocked. Further analyzing the crosstalk between autophagic and caspase activation in CRC cells, we found autophagy is essential for activation of caspase-8, and ATS switches to activate capase-8 via induction of autophagy when caspase-9 is inhibited. When apoptosis is totally blocked, HF-ATS switches to induce autophagic cell death. This scenario was then confirmed in studies of chemoresistance CRC cells with defective apoptosis. Our results indicate that HF-ATS induces cell death via interaction between apoptosis and autophagy in CRC cells. These results highlight the value of continued investigation into the potential use of this combination in cancer therapy.

## Introduction

Colorectal cancer (CRC) is a malignant cancer, responsible for 8 % of all cancer deaths in the world 1. In China, CRC is the fifth and fourth most commonly diagnosed cancer among men and women, respectively 2. Chemotherapy is the most common treatment for CRC, but the efficacies of currently used drugs are often limited by toxicity or acquired resistance, therefore there is the need of novel approaches for CRC treatment 3.

Combination therapy is an increasingly common and effective strategy for cancer treatment 4. Halofuginone (HF) and artemisinin (AST) are two active compounds derived from a Chinese traditional herb formula comprised of *Dichroa febrifuga* Lour (Changshan in Chinese) and *Artemisia carvifolia* (Qinghao in Chinese). The two compounds are used in the treatment of various diseases, including malaria and cancer 5, 6. Our previous study indicates that in CRC cell lines, HF-ATS synergistically arrests the cell cycle at the G1/G0 phase by upregulating p21^cip1^ and p27^kip1^^7^.

Besides cell cycle regulation, cell death is another basic cellular process that scientists are working on for developing potential cancer treatment reagent 8. In this study, we examined whether HF-ATS exhibited synergistic effect in inducing CRC cell death and revealed the underlying mechanism of action. Our specific hypothesis was that HF-ATS synergistically induces CRC cell death via induction of apoptosis and that induction may be associated with regulated autophagy. To test the hypothesis, we performed *in vitro* and *in vivo* experiments with 5-fluorouracil (5Fu)-resistant CRC cells and tumor tissues from a xenograft subcutaneous CRC model. Using these cells and tissues, we demonstrated that HF-ATS induces cell death in CRC cells by coordinating crosstalk between apoptosis and autophagy.

## Materials and methods

### Chemicals, antibodies, inhibitors

Halofuginonehy drobromide, 5-fluorouracil, Earle's Balanced Salt Solution (EBSS), chloroquine (CQ), propidium iodide (PI) and Pierce (R) BCA Protein Assay Kit were obtained from Sigma-Aldrich (Munich, Germany). Artemisinin and In situ BrdU-red DNA fragmentation (TUNEL) assay kit were purchased from Abcam (Cambridge, UK). Antibodies against cleaved capase-8, cleaved capase-9, cleaved caspase-3, cleaved PARP, SQSTM1/p62, LC3-II and β-Actin were purchased form Cell Signaling Technology (Danvers, MA, USA). HRP-goat anti-rabbit secondary antibody was purchased from Invitrogen (Carlsbad, CA, USA). Goat antimouse IgG-HRP secondary antibody and caspase-3 inhibitor (z-DEVD-fmk) were purchased from San Cruz Biotechnology (Santa Cruz, CA, USA). Caspase-8 inhibitor (z-IETD-fmk) and caspase-9 inhibitor (z-LEHD-fmk) were purchased from R&D Systems (Wiesbaden-Nordenstedt, Germany). FITC Annexin V Apoptosis Detection Kit I was obtained from BD Bioscience (San Jose, CA, USA).

### Cell culture

HCT116 and DLD-1 cells were purchased from American Type Culture Collection (Manassas, VA, USA). Cells were cultured in DMEM supplemented with 10 % FBS in a humidified atmosphere containing 5 % CO_2_ and 95 % air at 37 °C. The medium was changed every three days, and cells were passaged using 0.05 % trypsin/EDTA.

### Development of 5Fu-resistant HCT116 cells

HCT116 cells stably resistant to 5Fu were developed by exposing parental HCT116 cells to an initial dose of 5 μM and culturing surviving cells to a confluence of 80 % for three passages. The cells that survived initial 5Fu treatment were then exposed to 10 μM for three passages and then to 20 μM for three passages. Finally, the 5Fu concentration was increased to 100 μM. The surviving resistant cells were named HCT116/5Fu-R.

### Annexin V/PI staining

Cells were seeded in 6-well plates 24 h prior to HF treatment at a density of 5×10^5^ cells per well. After treatment with HF, ATS, or HF-ATS for 24 h, PI staining was performed to distinguish dead cells, in which plasma membranes become permeable regardless of the mechanism of death 9. For identification of apoptotic cells, Annexin V/PI double staining was performed. In the process of apoptosis, the membrane phospholipid phosphatidylserine (PS) is translocated from the inner to the outer leaflet of the plasma membrane, which precedes the loss of membrane integrity. Therefore, early apoptotic cells are Annexin V positive and PI negative, and late apoptotic cells are both Annexin V and PI positive 10.

### GFP-LC3-II translocation and imaging in living cells

Cells were transfected with pEGFP-LC3-II plasmids using lipofectamine 2000 (Invitrogen, 11668-019). One day after transfection, cells were treated with HF, ATS, or HF-ATS for 24 h, and then fixed. Cells were imaged for GFP using a Leica TCS SP8 (Leica) confocal microscope in the detected channel (excitation wavelength 488 nm, emission filter 500∼550 nm).

### Western blot analysis

Cells were treated with HF, ATS, or HF-ATS for 24 h, and whole cell lysates were then obtained by suspending the cells in lysis buffer. Following centrifugation at 13,500 rpm for 15 min at 4 °C, total protein concentration was measured using Pierce (R) BCA Protein Assay Kit, and 10 to 25 μg of protein was separated on 10 % SDS-PAGE and transferred to PVDF membranes. After blocking (5 % skim milk powder in TBST, 20) for 1 h at room temperature, the membrane was then incubated with primary antibody overnight at 4 °C. The membrane was incubated with secondary antibody for 1 h at room temperature. All antibodies were diluted in TBS-Tween 20 containing 5 % dry milk. The immune-reactive proteins were detected by enhanced chemiluminescence (ECL) using X-ray film and ECL reagent.

### *In vivo* animal study

As described in our previous study 7, HCT116 cells (5 × 10^6^ cells per mouse) were suspended in PBS and inoculated subcutaneously in the left flank of BALB/c nude mice.

After that, tumor growth was monitored regularly. Once tumors were palpable (~100 mm^3^), mice were divided at random into different groups, each group with 5 mice, treated as HF (daily i.p. 5 µg/kg of HF), ATS (daily i.p. 50 mg/kg of ATS), and HF-ATS (daily i.p. 5 µg/kg of HF and 50 mg/kg of ATS).

### Statistical analysis

Each experiment was performed at least three times. GraphPad Prism 5.0 software was used for statistical analysis.

## Results

### HF-ATS induces caspase-dependent apoptosis

**Figure 1:**
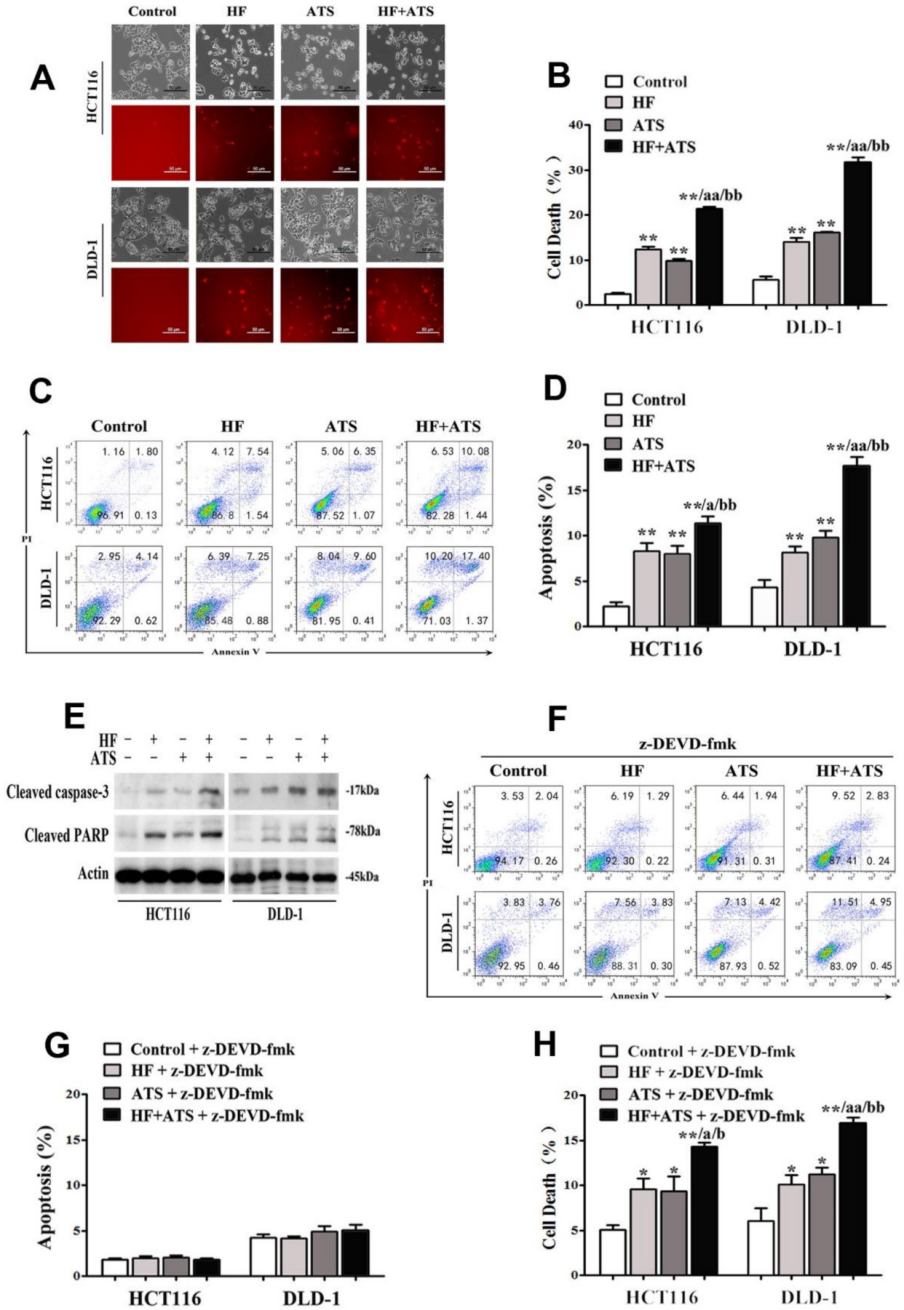
** HF-ATS synergistically induces caspase-dependent apoptosis. (A, B)** HF-ATS induces more cell death. In A, popidium iodide (PI) staining shows cell deaths, as seen through a light microscope, of HCT116 and DLD-1 cells treated with combination of HF (10 nM) and ATS (160 μM) for 24 h. Upper panel: phase-contrast; lower panel: PI staining. Scale bar = 50 μm. In B, cell death was quantified with flow cytometry. ** P < 0.01, compared with control group. aa P < 0.01, compared with HF. bb P < 0.01, compared with ATS. **(C, D)** HF-ATS induces increased apoptosis. In C, flow cytometry analyses of apoptosis in HCT116 and DLD-1 cells treated with combination of HF (10 nM) and ATS (160 μM) for 24 h. In D, apoptosis was quantified by Annexin V-FITC/PI staining coupled with flow cytometry. ** P < 0.01, compared with control group. a P < 0.05, compared with HF. bb P < 0.01, compared with ATS. **(E)** HF-ATS increases higher expression of cleaved caspase-3 and PARP in HCT116 and DLD-1 cells.** (F, G)** Apoptosis was blocked by caspase-3 inhibitor. In F, flow cytometry analyses of apoptosis regulated by combination of HF (10 nM) and ATS (160 μM) for 24 h in HCT116 and DLD-1 cells pretreated with caspase-3 inhibitor (z-DEVD-fmk). In G, apoptosis was quantified by Annexin V-FITC/PI staining coupled with flow cytometry. **(H)** Cell death was quantified by combination of HF (10 nM) and ATS (160 μM) for 24 h in HCT116 and DLD-1 cells pretreated with caspase-3 inhibitor (z-DEVD-fmk). * P < 0.05, ** P < 0.01, compared with control group. a P < 0.05, aa P < 0.01, compared with HF. b P < 0.05, bb P < 0.01, compared with ATS.

To examine the potential synergistic anti-CRC activity of HF-ATS, HCT116 and DLD-1 cells were cultured. As shown in **Figure 1A and 1B**, HF-ATS incurred a greater percentage of cell death compared with HF and ATS, which was consistent with our previous report 7. It is known that HF and ATS both can induce apoptosis in various cancer cells; therefore, we further investigated whether HF-ATS has a greater effect on apoptosis. As seen in **Figure 1C** and** 1D**, Annexin V-FITC/PI staining showed that the mono-treatments indeed induced apoptosis, and the induction of apoptosis was more prominent after the HF-ATS treatment. Consistently, HF and ATS markedly activated cleaved caspase-3 and PARP. Meanwhile, higher expression of cleaved caspase-3 and PARP resulted from HF-ATS treatment as shown by Western blot analysis (**Figure 1E**). To further dissect the involvement of caspase-3 in mediating the apoptosis under HF-ATS treatment, we used z-DEVD-fmk to mediate the inhibition of caspase-3. Although pretreatment of cells with z-DEVE-fmk for 1 h almost completely blocked apoptosis induced by HF, ATS or HF-ATS (**Figure 1F, 1G and S1**), it is interesting that HF-ATS still incurred a greater percentage of cell deaths compared with single-agent treatment (**Figure 1H and S2**).

### HF-ATS induces apoptosis through caspase-8 and caspase-9

**Figure 2:**
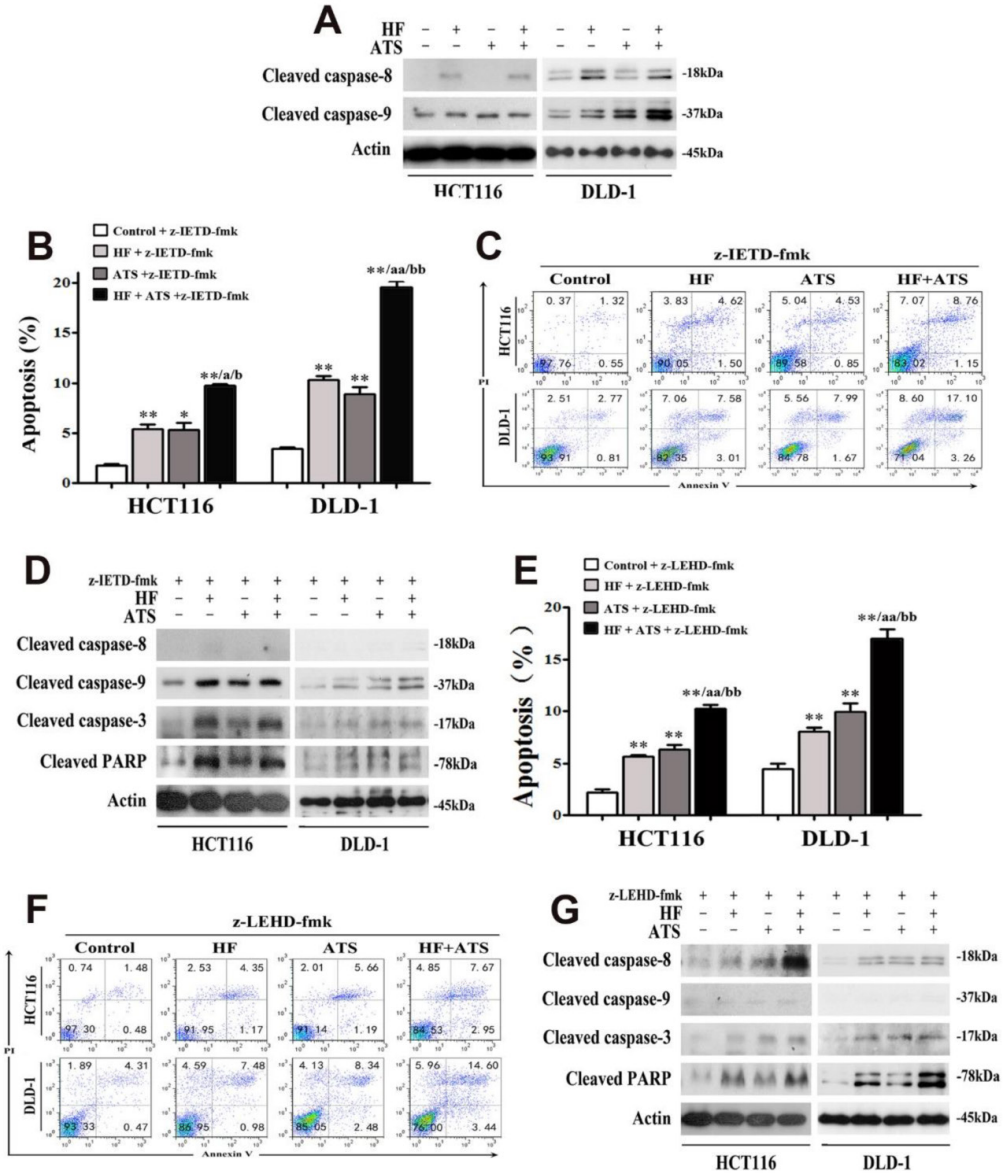
** Both caspase-8 and caspase-9 are involved in HF-ATS-induced apoptosis. (A)** Expression of cleaved capase-8 and caspase-9 in HCT116 and DLD-1 cells treated with combination of HF (10 nM) and ATS (160 μM) for 24 h. **(B, C)** For caspase 8 inhibitor (z-IETD-fmk) pretreated cells, HF-ATS increased the number of apoptotic cells. In B, flow cytometry analyses of apoptosis regulated by combination of HF (10 nM) and ATS (160 μM) for 24 h in HCT116 and DLD-1 cells pretreated with caspase-8 inhibitor (z-IETD-fmk). In C, apoptosis was quantified with flow cytometry. * P < 0.05, ** P < 0.01, compared with control group. a P < 0.05, aa P < 0.01, compared with HF. b P < 0.05, bb P < 0.01, compared with ATS. **(D)** Expression of cleaved capase-8, capase-9, caspase-3 and PARP regulated by combination of HF (10 nM) and ATS (160 μM) for 24 h in HCT116 and DLD-1 cells pretreated with caspase-8 inhibitor (z-IETD-fmk). **(E, F)** For caspase-9 inhibitor (z-LEHD-fmk) pretreated cells, HF-ATS increased the number of apoptotic cells. In E, flow cytometry analyses of apoptosis regulated by combination of HF (10 nM) and ATS (160 μM) for 24 h in HCT116 and DLD-1 cells pretreated with caspase-9 inhibitor (z-LEHD-fmk). In F, apoptosis was quantified with flow cytometry. ** P < 0.01, compared with control group. aa P < 0.01, compared with HF. bb P < 0.01, compared with ATS.** (G)** Expression of cleaved capase-8, capase-9, caspase-3 and PARP regulated by combination of HF (10 nM) and ATS (160 μM) for 24 h in HCT116 and DLD-1 cells pretreated with caspase-9 inhibitor (z-LEHD-fmk).

Different caspases are activated at the initiation and execution phases of apoptosis. Briefly, caspase-8 and caspase-9 are two essential triggers separately involved in extrinsic and intrinsic apoptosis, respectively; both cleave and activate downstream caspase-3 then induce apoptosis 11. To determine which caspase pathways are essential for induction of apoptosis by HF, ATS or HF-ATS, we performed Western blot and found that ATS increased only the level of cleaved caspase-9, but HF and HF-ATS increased the level of cleaved caspase-9 and caspase-8 (**Figure 2A**). To further elucidate the role of caspase 8 and caspase 9 in inducing apoptosis under HF-ATS treatment, different caspase inhibitors were administered. In cells pretreated with caspase-8 inhibitor (z-IETD-fmk), HF and ATS both induced apoptosis; and HF-ATS increased the count of apoptotic cells to a greater extent than single-agent treatments (**Figure 2B** and** 2C**). Furthermore, HF and ATS both increased the level of cleaved caspase-3 and PARP by caspase-9 activation when caspase-8 was inhibited (**Figure 2D**).

In contrast, ATS and HF still induced apoptosis in CRC cells after they had been pretreated with caspase-9 inhibitor (z-LEHD-fmk) (**Figure 2E and 2F**). More interesting, activation of caspase-8 was involved in induction of apoptosis by ATS when caspase-9 was inhibited. HF and HF-ATS also induced apoptosis by activating caspase-3 via upstream caspase-8 activation (**Figure 2G**). As predicted, after pretreatment with the combination of caspase-8 and caspase-9 inhibitors (z-IETD-fmk + z-LEHD-fmk) in HCT116 and DLD-1 cells, these two caspases were inhibited and none of the treatments (HF, ATS and HF-ATS) induced apoptosis (**Figure S3 and S4**).

### HF-ATS induces autophagy-dependent activation of caspase-8

**Figure 3:**
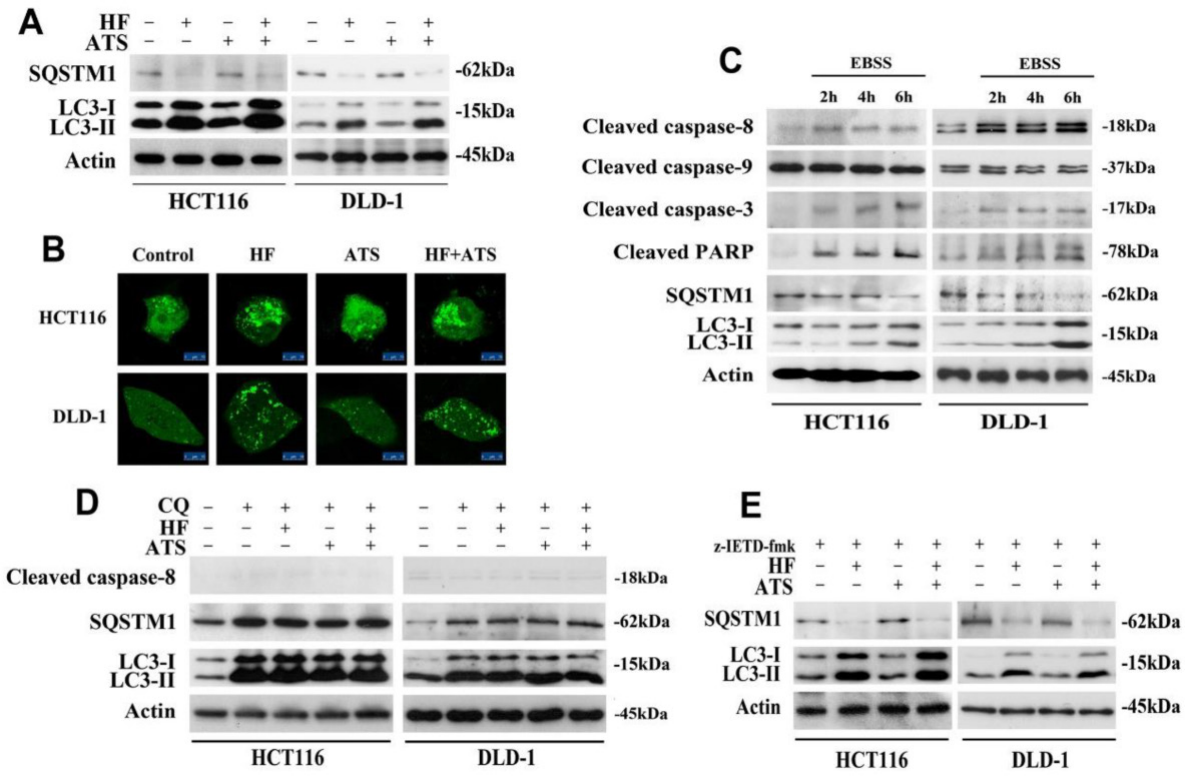
** HF-ATS induces autophagy-dependent activation of caspase-8. (A)** Expression of SQSTM1 and LC3-II in HCT116 and DLD-1 cells treated with combination of HF (10 nM) and ATS (160 μM) for 24 h.** (B)** Accumulation of cytoplasmic GFP-LC3-II dots in HCT116 and DLD-1 cells treated with combination of HF (10 nM) and ATS (160 μM) for 24 h. Distribution of GFP-LC3-II was examined by confocal microscope. Scale bar = 50 μm. **(C)** Expression of cleaved capase-8, capase-9, caspase-3, PARP, SQSTM1 and LC3-II in HCT116 and DLD-1 cells cultured in EBSS medium. **(D)** Expression of cleaved capase-8, SQSTM1 and LC3-II regulated by combination of HF (10 nM) and ATS (160 μM) for 24 h in HCT116 and DLD-1 cells co-treated with autophagic inhibitor CQ. **(E)** Expression of SQSTM1 and LC3-II regulated by combination of HF (10 nM) and ATS (160 μM) for 24 h in HCT116 and DLD-1 cells pretreated with caspase-8 inhibitor (z-IETD-fmk).

Recently, the crosstalk and molecular mechanisms of apoptosis and autophagy have been extensively studied because apoptosis and autophagy are often regulated by similar pathways and engage common sub-cellular sites 12. Firstly, to determine whether HF-ATS kills cancer cells by autophagy, we performed Western blot. This enabled us to examine the expression of SQSTM1/p62 and microtubule-associated protein 1A/1B-light chain 3-II (LC3-II), which play critical roles in the process of autophagy 13. As shown in **Figure 3A**, HF and HF-ATS reduced the expression of SQSTM1, which is a substrate of autophagic degradation. Meanwhile, HF and HF-ATS increased the expression of LC3-II, which is converted from LC3-I. However, ATS couldn't change the expression of these two proteins. Consistently, as shown in **Figure 3B**, compared with control group, HF and HF-ATS induced accumulation of GFP-LC3 dots in cytoplasm, while ATS could not. These results suggest that HF but not ATS can affect autophagy in CRC cells.

To determine the relationship between apoptosis and autophagy, nutrient-deprived medium (EBSS) was employed. When CRC cells were cultured in EBSS, over time, SQSTM1 gradually, clearly reduced while LC3-II increased. Notably, with induction of autophagy, caspase-8, as well as downstream caspase-3 and PARP, were cleaved and activated, but there was no effect on caspase-9 (**Figure 3C**). These results suggest that autophagy is essential for activation of caspase-8 and that, after activation, caspase-8 then induces apoptosis in CRC cells.

To further analyze the role of autophagy in caspase-8 activation, the autophagic inhibitor chloroquine (CQ) was employed. In cells co-treated with CQ and HF, ATS or HF-ATS, autophagy was inhibited, and all treatments had no effect on activation of caspase-8 (**Figure 3D**). We then analyzed autophagic markers after caspase-8 had been inhibited. As shown in **Figure 3E**, HF or HF-ATS reduced SQSTM1 and increased LC3-II. This suggests that autophagy is an upstream factor for caspase-8 activation, which then induces apoptosis; and HF-ATS activates caspase-8 through induction of autophagy.

### HF-ATS induces autophagic cell death when apoptosis is inhibited

**Figure 4:**
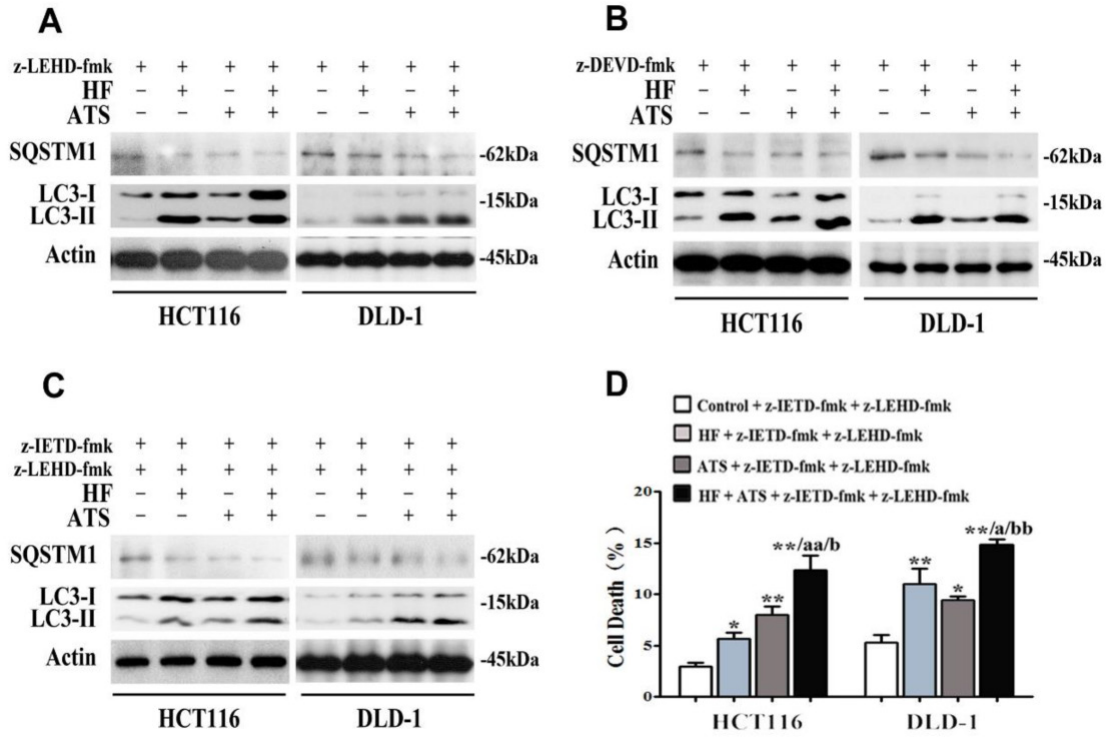
** HF-ATS switches to autophagy when apoptosis is blocked. (A)** Expression of cleaved SQSTM1 and LC3-II regulated by combination of HF (10 nM) and ATS (160 μM) for 24 h in HCT116 and DLD-1 cells pretreated with caspase-9 inhibitor (z-LEHD-fmk). **(B)** Expression of cleaved SQSTM1 and LC3-II regulated by combination of HF (10 nM) and ATS (160 μM) for 24 h in HCT116 and DLD-1 cells pretreated with caspase-3 inhibitor (z-DEVD-fmk). **(C)** Expression of cleaved SQSTM1 and LC3-II regulated by combination of HF (10 nM) and ATS (160 μM) for 24 h in HCT116 and DLD-1 cells pretreated with caspase-8 and caspase-9 inhibitors together (z-IETD-fmk + z-LEHD-fmk). **(D)** Cell death was quantified by combination of HF (10 nM) and ATS (160 μM) for 24 h in HCT116 and DLD-1 cells pretreated with caspase-8 and caspase-9 inhibitors together (z-IETD-fmk + z-LEHD-fmk). * P < 0.05, ** P < 0.01, compared with control group. a P < 0.05, aa P < 0.01, compared with HF. b P < 0.05, bb P < 0.01, compared with ATS.

Our data showed that ATS switched to induce apoptosis via caspase-8, when caspase-9 was blocked (**Figure 2D** and **2E**). Because autophagy is essential for caspase-8 activation (**Figure 3C**), we hypothesized that ATS may promote autophagy to induce apoptosis via the caspase-8 activation when caspase-9 is inhibited. In order to verify that, caspase-9 inhibitor (z-LEHD-fmk) was used again. As shown in **Figure 4A**, ATS, HF and HF-ATS reduced the expression of SQSTM1 and increased the LC3-II in cells pretreated with caspase-9 inhibitor. This phenomenon demonstrates ATS switches to induction of autophagy when caspase-9 is blocked; it can explain why ATS still induces apoptosis via caspase-8 activation as shown in **Figure 2F** and **2G.**

It's interesting that HF-ATS increased the percentage of cell death when apoptosis was completely blocked (**Figure 1H**). Although the role of autophagy in cancer development is still a conundrum, therapeutic modulation of autophagy has been considered as a potential approach for cancer treatment 14. Therefore, we hypothesize that when apoptosis is blocked, HF-ATS may switch apoptotic cell death to autophagic cell death. To detect any crosstalk between apoptosis and autophagy regulated by HF-ATS, cells were pretreated with a combination of caspase-8 and caspase-9 inhibitors (z-IETD-fmk+z-LEHD-fmk) or caspase-3 inhibitor (z-DEVD-fmk) alone to block apoptosis. Results showed that HF, ATS or HF-ATS reduced SQSTM1 and increased LC3-II (**Figure 4B and 4C**), indicating that HF, ATS or HF-ATS stimulated autophagy when apoptosis pathways were inhibited. Moreover, as shown in **Figure 4D**, HF, ATS and HF-ATS still induced cell death when caspase-8 and caspase-9 both were blocked; and cell death was also observed in all treatments upon caspase-3 inhibition (**Figure 1H**), which demonstrates that HF, ATS or HF-ATS induces cell death through induction of autophagy.

### HF-ATS induces autophagic death in apoptosis-defective cells

**Figure 5:**
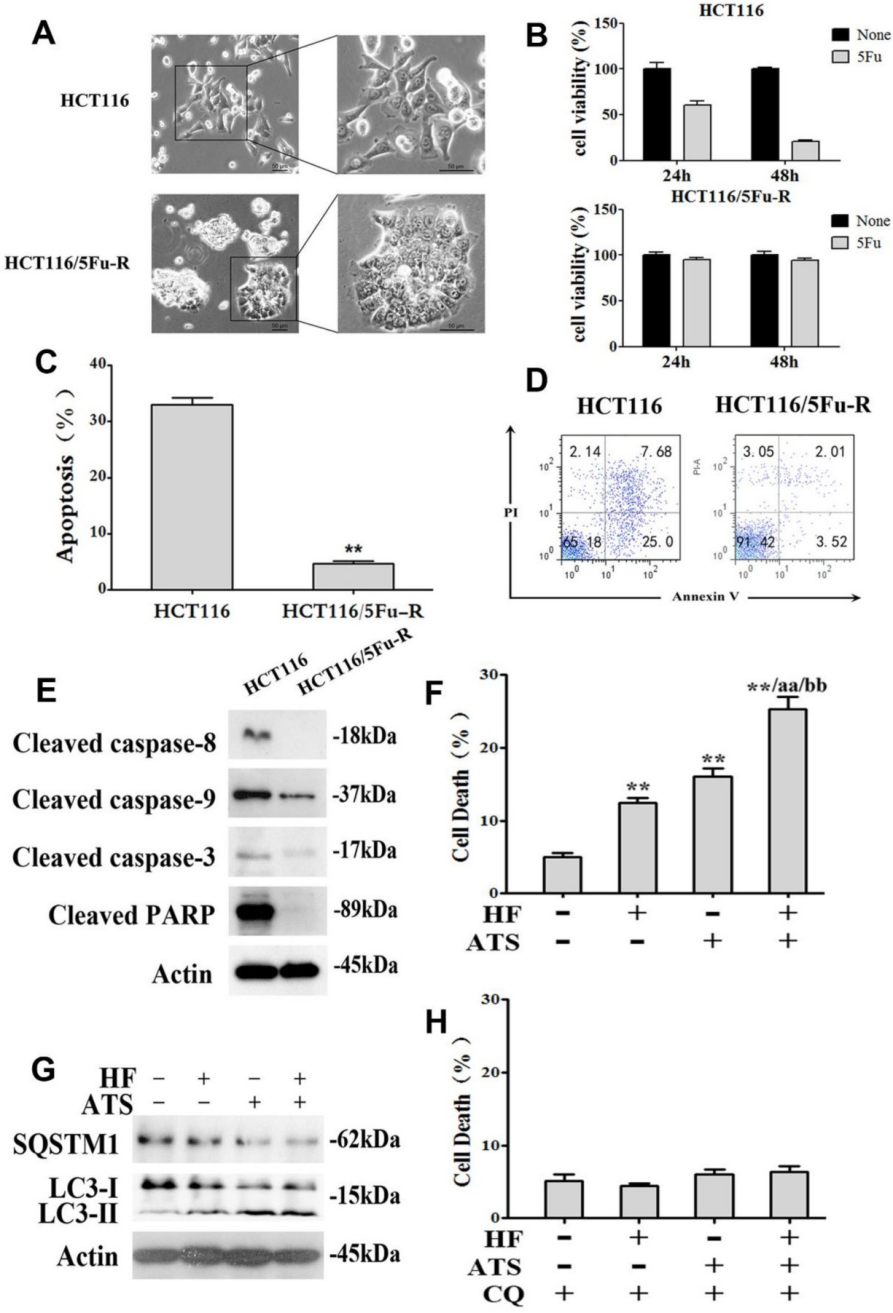
** HF-ATS induces autophagic death in 5Fu resistant HCT116 cells. (A)** Morphologic analysis comparing parental and 5Fu resistant HCT116 cells. Scale bar = 50 μm. **(B)** Cell viability between parental and 5Fu resistant HCT116 cells by MTT assay, which were exposed to 100 mM 5Fu for 24 h and 48 h. **(C, D)** 5Fu resistant HCT116 cells escape apoptosis. In C, apoptosis was quantified with flow cytometry. In D, flow cytometry analyses of apoptosis in parental and 5Fu resistant HCT116 cells, both exposed to 100 mM 5Fu for 24 h. **(E)** Expression of cleaved caspase-8, caspase-9, caspase-3 and PARP regulated by 100 mM 5Fu in 5Fu resistant HCT116 cells. **(F)** Cell death was quantified by combination of HF (10 nM) and ATS (160 μM) for 24 h in 5Fu resistant HCT116 cells. * P < 0.05, ** P < 0.01, compared with control group. a P < 0.05, aa P < 0.01, compared with HF. b P < 0.05, bb P < 0.01, compared with ATS. **(G)** Expression of cleaved SQSTM1 and LC3-II regulated by combination of HF (10 nM) and ATS (160 μM) for 24 h in 5Fu resistant HCT116 cells. **(H)** Cell death was quantified by combination of HF (10 nM) and ATS (160 μM) for 24 h in 5Fu resistant HCT116 cells co-treated with autophagic inhibitor CQ.

Resistance to chemotherapy is a common phenomenon in cancer treatment and results in therapeutic failure 15. Cancer cells can develop chemoresistance and escape apoptotic death with some specific machineries after long-term drug exposure 16. To investigate whether HF-ATS can induce non-apoptotic death in chemoresistant cells which have defective apoptosis, we developed 5Fu-resistant HCT116 cells (HCT116/5Fu-R). As shown in **Figure 5A**, HCT116/5Fu-R cells had a markedly different appearance under the light microscope compared with parental HCT116 cells, including loss of cell polarity causing a spindle-cell morphology and increased intercellular separation signifying loss of intercellular adhesion. *In vitro* viability of parental and 5Fu-resistant HCT116 cells which had been exposed to a clinically relevant dose (i.e., 100 μM) of 5Fu were compared by MTT assay. As shown in **Figure 5B**, parental cells were sensitive to 5Fu, with only 60.4 % and 20.4 % of viable cells remaining after exposure for 24 h and 48 h, respectively. With HCT116/5Fu-R cells, as expected, 94.9 % and 94.2 % of viable cell remained after 24 h and 48 h, respectively. Moreover, compared with parental cells exposed to 5Fu, HCT116/5Fu-R cells were found to escape apoptosis through inactivating upstream caspase-8 and caspase-9 and downstream caspase-3 pathways (**Figure 5C, 5D and 5E**). Interestingly, we found that HF-ATS still induced more HCT116/5Fu-R cell deaths compared with HF or ATS, as shown by PI staining (**Figure 5F and S6**). This staining revealed that induction of chemoresistant cell death was via mechanism(s) different from apoptosis. Subsequent study showed that, when apoptosis was defective, the death of chemoresistant cells was associated with induction of autophagy by HF, ATS and HF-ATS (**Figure 5G**), which was prevented after co-treated with autophagic inhibitor CQ (**Figure 5H and S7**).

### HF-ATS coordinates apoptosis and autophagy *in vivo*

**Figure 6:**
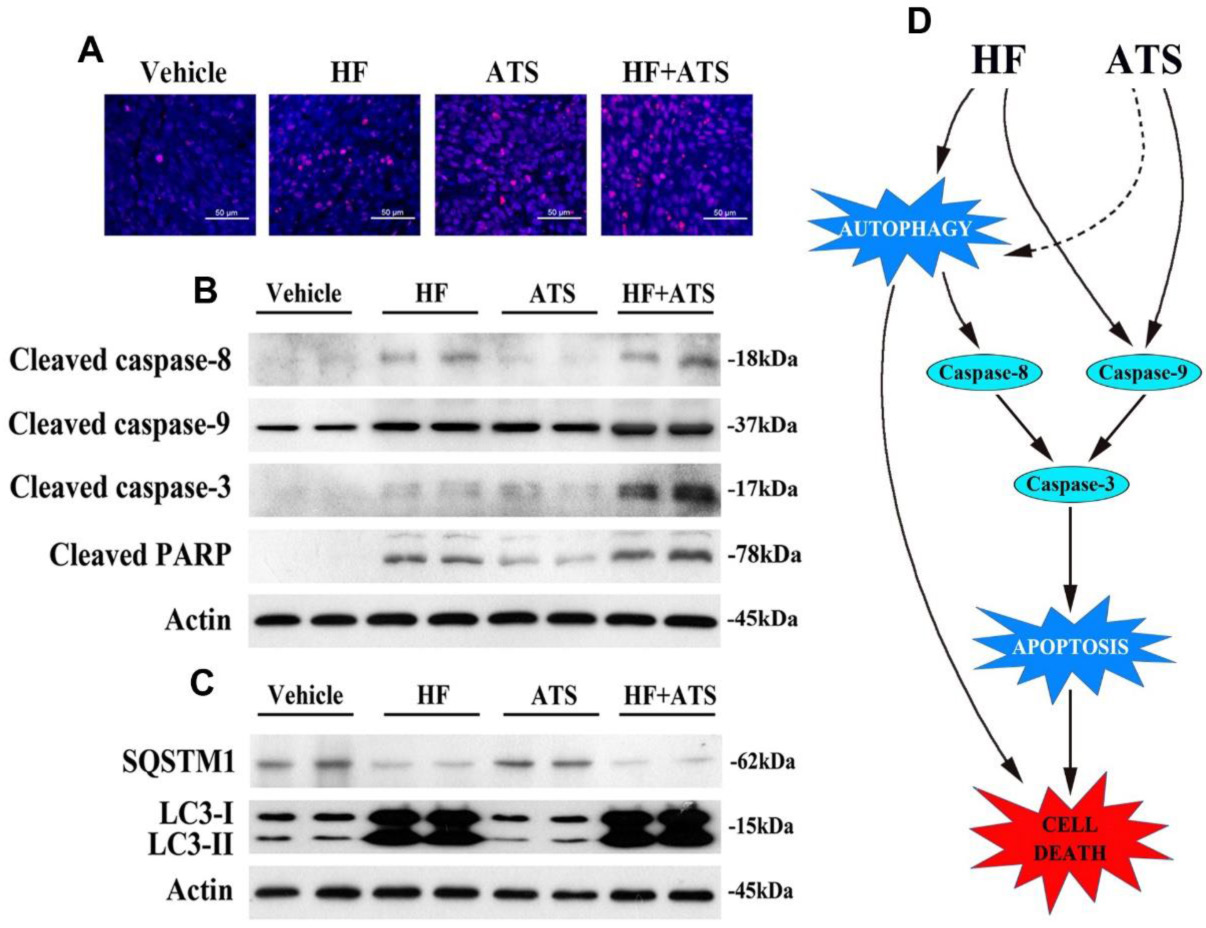
** HF-ATS coordinates apoptosis and autophagy in xenograft nude mice. (A)** TUNEL staining of paraffin-embedded 5 micron-thick tumor sections from HCT116 xenograft-bearing nude mice. Scale bar = 50 μm. (B) Expression levels of cleaved aspase-8, caspase-9, caspase-3 and PARP in xenograft tumors. **(C)** Expression levels of SQSTM1 and LC3-II in xenograft tumors. **(D)** Proposed mechanism of cell death regulated by the HF-ATS in CRC. HF-ATS synergistically induces caspase-dependent apoptosis through activation of caspase-9 and caspase-8. Autophagy is essential for activation of caspase-8, which can be induced by HF or HF-ATS. However, ATS switches to activate capase-8 via induction of autophagy when caspase-9 is inhibited (dotted line). When apoptosis is blocked, HF-ATS switches to induce autophagic death of CRC cells.

In our previous study, nude mice were injected subcutaneously with HCT116 cells and then co-administrated with HF and ATS. Results showed the tumor volumes and masses in mice exposed to HF-ATS were significantly lower than those in mice treated with HF and ATS. This means HF-ATS showed synergistic inhibition of tumor growth *in vivo* 7. In order to investigate cell death via apoptosis and autophagy, we isolated tumor tissues from nude mice co-administrated with HF, ATS, and HF-ATS. As shown in **Figure 6A**, HF or ATS induced modest apoptosis as shown by TUNEL staining, and HF-ATS induced stronger apoptosis in tumor tissues, which was consistent with the *in vitro* data (**Figure 1C and 1D**). Next, Western blot and immunofluorescence staining were preformed to detect the expression levels of apoptotic markers in the tumors from different groups. Although the levels of cleaved caspase-8 and caspase-9 were increased in HF and HF-ATS groups, the change was more significant in the latter group. In the ATS group, only cleaved caspase-9 was increased (**Figure 6B and S8**). In addition, autophagic markers were also analyzed. As shown in **Figure 6C and S9**, a reduction of SQSTM1 and an increase of LC3-II were observed in HF and HF-ATS groups. In the ATS group, the two autophagic markers had no change compared with the control group. In summary, these results indicate that the synergistic inhibition of tumor growth by HF-ATS is associated with induction of cell death via apoptosis and autophagy, consistent with the observations from our cell culture experiments.

## Discussion

CRC is a major health concern worldwide. The current treatments have adverse side effects, and drug resistance leads to unsatisfactory treatment results. As a result, researchers have been seeking to develop new approaches to treatment 17. In our previous study, we demonstrated that HF-ATS has synergistic effects on CRC that it induces cell cycle arrest at the G1/G0 phase in cancers, and that p21^cip1^ and p27^kip1^ are two key factors in this cell cycle arrest 7.

Besides modulation of the cell cycle, induction of cell death is another anticancer strategy 18. Accumulating evidence shows that cell death plays a key role in ultimate decisions of cancer cell fate 19. Apoptosis is a classic form of cell death, and it can functionally interact with the stress-responsive process, autophagy, to determine the fate of cancerous cells 20. In this study, we found that treatment with HF or ATS induced apoptosis in CRC cells, and this effect was consistent with previous reports 21-24. Importantly, treatment with HF-ATS increased the count of apoptotic cells compared with either agent alone. Interestingly, although ATS alone had no effect on autophagy in CRC cells, we found that HF, or HF-ATS induced autophagy.

Caspases are a family of protease enzymes playing essential roles in apoptosis. Activated caspase-8 and caspase-9 are separate and distinct, but both initiate apoptosis directly by cleaving and activating executioner caspase-3 25. In our studies, we found that ATS activated caspase-3 through activation of caspase-9, while HF activated caspase-3 in association with the activation of both caspase-8 and caspase-9. These data suggest that caspase-8 and caspase-9 are key factors involved in induction of apoptosis by HF-ATS.

Cancer cells often develop apoptosis-evading mechanisms which contribute to their malignancy and drug-resistance capability. Importantly, deficiency of either caspase-8-mediated extrinsic apoptotic pathway or caspase-9-mediated intrinsic pathway alone is not sufficient to ablate CRC apoptosis induced by HF-ATS. When caspase-8 was inhibited, HF and ATS still induced HCT116 and DLD-1 cells to undergo apoptosis; and HF-ATS induced a greater percentage of cells to become apoptotic compared with single agent treatment (**Figure 2B and 2C**). Conversely, ATS, as well as HF, induced cells to undergo apoptosis when pretreated with caspase-9 inhibitor; and activation of caspase-8 was involved in this effect (**Figure 2E, 2F and 2G**). On the other hand, inhibition of both caspase-8 and caspase-9 completely abolished the induction of apoptosis by HF, ATS, or HF-ATS.

Under certain specific conditions, autophagy may help to promote apoptosis 26. Excessive autophagy has also been shown to degrade the cytoplasm beyond recovery, leading to 'autophagic cell death' 27. In our study, we utilized EBSS medium to induce autophagy in CRC cells and found that caspase-8, not caspase-9, was cleaved and activated with induction of autophagy, which then induced apoptosis via activating downstream caspase-3. When cells were co-treated with autophagic inhibitor CQ, autophagy was blocked and all treatments had no effect on activation of caspase-8. Furthermore, when apoptosis was completely blocked through inhibition of both caspase-8 and caspase-9, cells switched to non-apoptotic cell death by HF-ATS (**Figure 6D**).

In order to verify the non-apoptoticc cell death by HF-ATS, we developed HCT116/5Fu-R cells, which have been exposed long-term to anticancer drugs and have defects in apoptosis 28. We found that HF-ATS-induced HCT116/5Fu-R cell death is associated with induction of autophagy. Therefore, these results demonstrate that HF-ATS induces not only apoptosis but also autophagic cell death in CRC.

In summary, our findings indicate that in CRC cells treated with HF-ATS, there is a coordinate crosstalk between apoptosis and autophagy which eventually leads to cell death. This work suggests the combinational drugs from this Chinese traditional herb formula can be a novel therapeutic strategy for the treatment of CRC.

## Supplementary Material

Supplementary figures.

## References

[1] Shin A, Jung KW, Won YJ (2013). Colorectal cancer mortality in Hong Kong of China, Japan, South Korea, and Singapore. World journal of gastroenterology.

[2] Chen W, Zheng R, Baade PD, Zhang S, Zeng H, Bray F (2016). Cancer statistics in China, 2015. CA Cancer J Clin.

[3] Housman G, Byler S, Heerboth S, Lapinska K, Longacre M, Snyder N (2014). Drug resistance in cancer: an overview. Cancers.

[4] Sun Y, Sheng Z, Ma C, Tang K, Zhu R, Wu Z (2015). Combining genomic and network characteristics for extended capability in predicting synergistic drugs for cancer. Nat Commun.

[5] Pines M (2014). Halofuginone for fibrosis, regeneration and cancer in the gastrointestinal tract. World journal of gastroenterology.

[6] Chaturvedi D, Goswami A, Saikia PP, Barua NC, Rao PG (2010). Artemisinin and its derivatives: a novel class of anti-malarial and anti-cancer agents. Chemical Society reviews.

[7] Chen G, Gong R, Shi X, Yang D, Zhang G, Lu A (2016). Halofuginone and artemisinin synergistically arrest cancer cells at the G1/G0 phase by upregulating p21Cip1 and p27Kip1. Oncotarget.

[8] Ouyang L, Shi Z, Zhao S, Wang FT, Zhou TT, Liu B (2012). Programmed cell death pathways in cancer: a review of apoptosis, autophagy and programmed necrosis. Cell proliferation.

[9] Gao M, Monian P, Quadri N, Ramasamy R, Jiang X (2015). Glutaminolysis and Transferrin Regulate Ferroptosis. Mol Cell.

[10] Vermes I, Haanen C, Steffens-Nakken H, Reutelingsperger C (1995). A novel assay for apoptosis. Flow cytometric detection of phosphatidylserine expression on early apoptotic cells using fluorescein labelled Annexin V. Journal of immunological methods.

[11] Andersen MH, Becker JC, Straten P (2005). Regulators of apoptosis: suitable targets for immune therapy of cancer. Nat Rev Drug Discov.

[12] Nikoletopoulou V, Markaki M, Palikaras K, Tavernarakis N (2013). Crosstalk between apoptosis, necrosis and autophagy. Biochimica et biophysica acta.

[13] Tanida I, Waguri S (2010). Measurement of autophagy in cells and tissues. Methods Mol Biol.

[14] Rubinsztein DC, Codogno P, Levine B (2012). Autophagy modulation as a potential therapeutic target for diverse diseases. Nat Rev Drug Discov.

[15] Luqmani YA (2005). Mechanisms of drug resistance in cancer chemotherapy. Medical principles and practice: international journal of the Kuwait University, Health Science Centre.

[16] Pommier Y, Sordet O, Antony S, Hayward RL, Kohn KW (2004). Apoptosis defects and chemotherapy resistance: molecular interaction maps and networks. Oncogene.

[17] Tao C, Sun J, Zheng WJ, Chen J, Xu H (2015). Colorectal cancer drug target prediction using ontology-based inference and network analysis. Database: the journal of biological databases and curation. 2015.

[18] Pucci B, Kasten M, Giordano A (2000). Cell cycle and apoptosis. Neoplasia.

[19] Fuchs Y, Steller H (2011). Programmed cell death in animal development and disease. Cell.

[20] Velentzas AD, Nezis IP, Stravopodis DJ, Papassideri IS, Margaritis LH (2007). Apoptosis and autophagy function cooperatively for the efficacious execution of programmed nurse cell death during Drosophila virilis oogenesis. Autophagy.

[21] Chen GQ, Tang CF, Shi XK, Lin CY, Fatima S, Pan XH (2015). Halofuginone inhibits colorectal cancer growth through suppression of Akt/mTORC1 signaling and glucose metabolism. Oncotarget.

[22] Jin ML, Park SY, Kim YH, Park G, Lee SJ (2014). Halofuginone induces the apoptosis of breast cancer cells and inhibits migration via downregulation of matrix metalloproteinase-9. International journal of oncology.

[23] Jia J, Qin Y, Zhang L, Guo C, Wang Y, Yue X (2016). Artemisinin inhibits gallbladder cancer cell lines through triggering cell cycle arrest and apoptosis. Mol Med Rep.

[24] Singh NP, Lai HC (2004). Artemisinin induces apoptosis in human cancer cells. Anticancer research.

[25] McIlwain DR, Berger T, Mak TW (2013). Caspase functions in cell death and disease. Cold Spring Harbor perspectives in biology.

[26] Young MM, Takahashi Y, Khan O, Park S, Hori T, Yun J (2012). Autophagosomal membrane serves as platform for intracellular death-inducing signaling complex (iDISC)-mediated caspase-8 activation and apoptosis. The Journal of biological chemistry.

[27] Marino G, Niso-Santano M, Baehrecke EH, Kroemer G (2014). Self-consumption: the interplay of autophagy and apoptosis. Nat Rev Mol Cell Biol.

[28] De Angelis PM, Svendsrud DH, Kravik KL, Stokke T (2006). Cellular response to 5-fluorouracil (5-FU) in 5-FU-resistant colon cancer cell lines during treatment and recovery. Molecular cancer.

