# Supplementary material for: Cell death mechanisms induced by synergistic effects of halofuginone and artemisinin in colorectal cancer cells
Source: Int J Med Sci. 2022 Jan 1;19(1):175–85. doi: 10.7150/ijms.66737 (PMC8692125; doi:10.7150/ijms.66737)
Supplement: Supplementary file 1 — Supplementary figures. [file ijmsv19p0175s1.pdf]

Supplementary Figure Legends

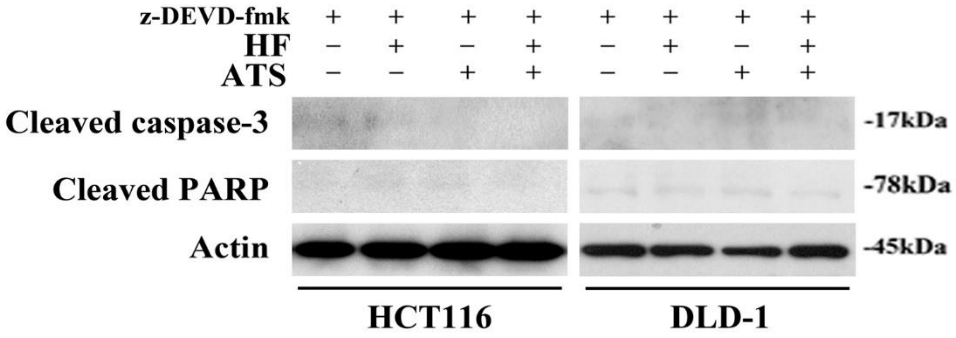

Supplementary Figure 1. Caspase-3 inhibitor (z-DEVD-fmk) blocks HF-ATS induced expression of cleaved caspase-3 and PARP in HCT116 and DLD-1 cells. HF:10 nM; ATS: 160 μM.

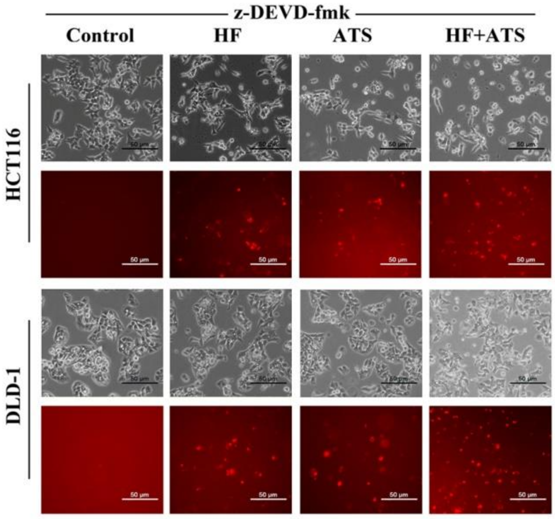

Supplementary Figure 2. PI staining analysis of cell death regulated by combination of HF (10 nM) and ATS (160 μM) for 24 h in HCT116 and DLD-1 cells pretreated with caspase-3 inhibitor (z-DEVD-fmk). Upper panel: phase-contrast, lower panel: PI staining. Scale bar = 50 μm.

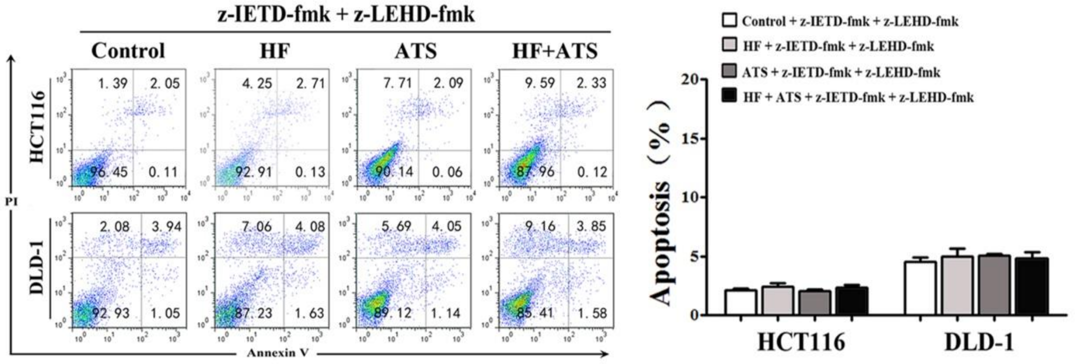

Supplementary Figure 3. Flow cytometry (right panel) and histogram (left panel) analyses of apoptosis regulated by combination of HF (10 nM) and ATS (160 μM) for 24 h in HCT116 and DLD-1 cell pretreated with caspase-8 and caspase-9 inhibitors together (z-IETD-fmk + z-LEHD-fmk).

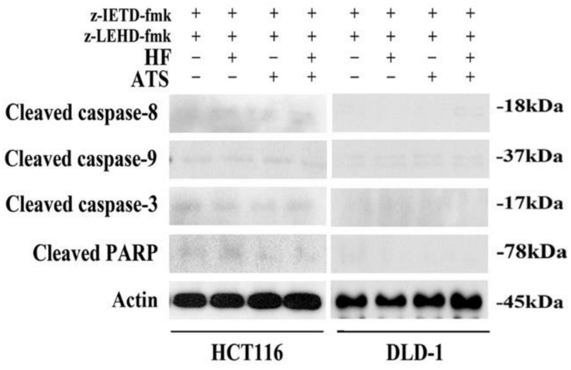

Supplementary Figure 4. Expression of cleaved capase-8, capase-9, caspase-3 and PARP regulated by combination of HF (10 nM) and ATS (160 μM) for 24 h in HCT116 and DLD-1 cells pretreated with caspase-8 and caspase-9 inhibitors together (z-IETD-fmk + z-LEHD-fmk).

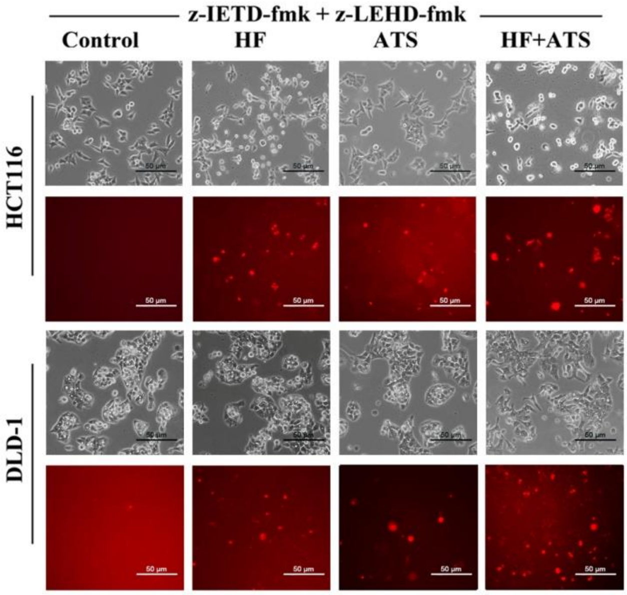

Supplementary Figure 5. PI staining analysis of cell death regulated by combination of HF (10 nM) and ATS (160 μM) for 24 h in HCT116 and DLD-1 cells pretreated with caspase-8 and caspase-9 inhibitors together (z-IETD-fmk + z-LEHD-fmk). Upper panel: phase-contrast, lower panel: PI staining. Scale bar = 50 μm.

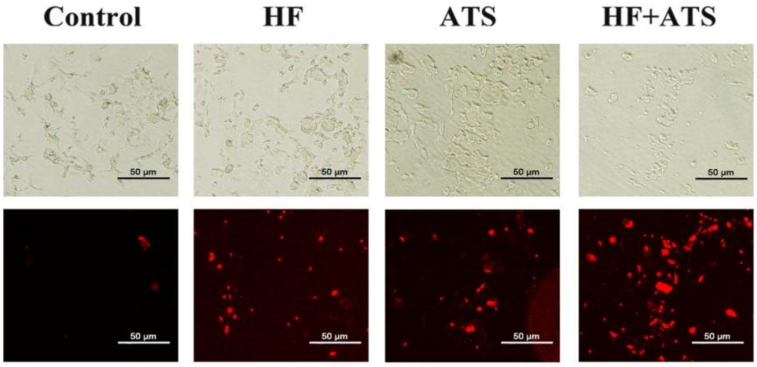

Supplementary Figure 6. PI staining analysis of cell death regulated by combination of HF (10 nM) and ATS (160 μM) for 24 h in 5Fu resistant HCT116 cells. Upper panel: phase-contrast, lower panel: PI staining. Scale bar = 50 μm

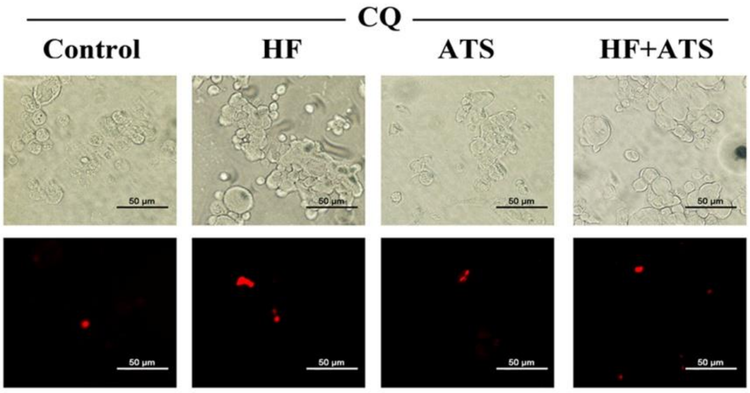

Supplementary Figure 7. PI staining analysis of cell death regulated by combination of HF (10 nM) and ATS (160 μM) for 24 h in 5Fu resistant HCT116 cells co-treated with autophgic inhibitor CQ. Upper panel: phase-contrast, lower panel: PI staining. Scale bar = 50 μm

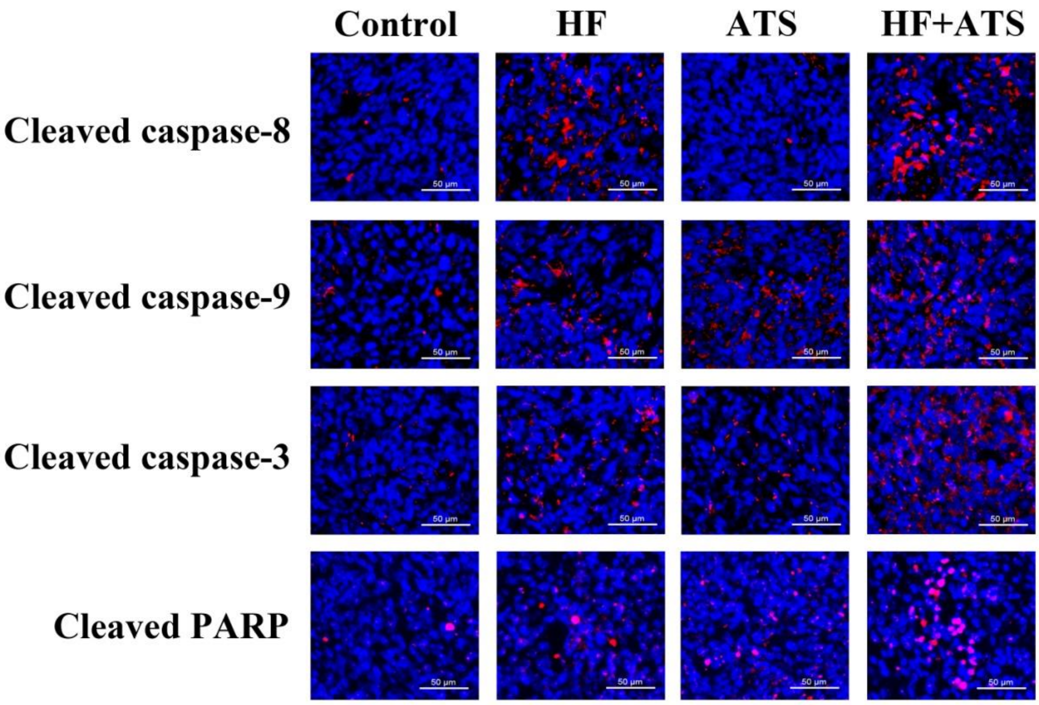

Supplementary Figure 8. Immunofluorescence staining for cleaved caspase-8, caspase-9 and caspase-3 in xenograft tumors. Scale bar = 50 μm.

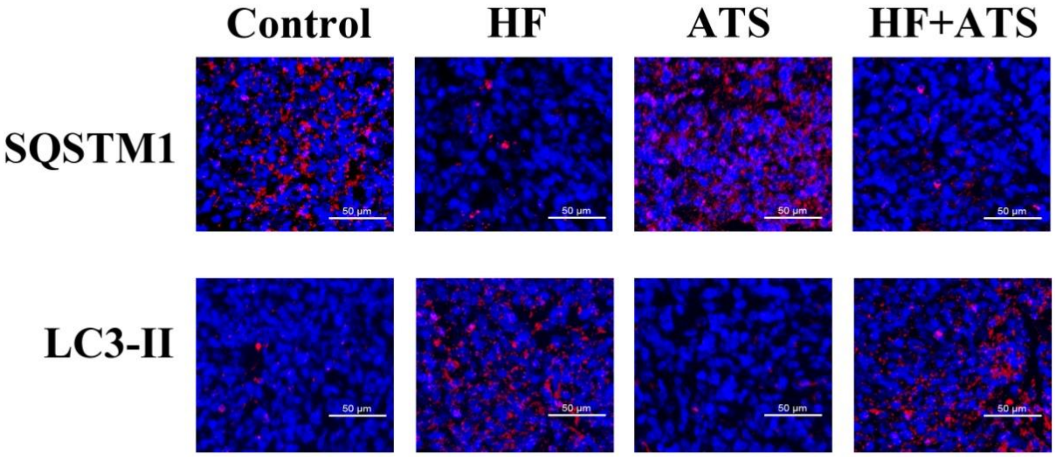

Supplementary Figure 9. Immunofluorescence staining for cleaved SQSTM1 and LC3-II in xenograft tumors. Scale bar = 50 μm.
